# Biphasic small bowel perforation after blunt abdominal trauma: a case report of delayed ischaemic progression and re-perforation

**DOI:** 10.1093/jscr/rjag450

**Published:** 2026-06-10

**Authors:** Martin Larisch, Jerome Martin Laurence, Basavaraj Mundasad, Sanjaya Karunaratne

**Affiliations:** General Surgery Department, Armidale Rural Referral Hospital, Hunter New England Health Services, 226 Rusden Street, Armidale NSW 2350, Australia; General Surgery Department, Armidale Rural Referral Hospital, Hunter New England Health Services, 226 Rusden Street, Armidale NSW 2350, Australia; Royal Prince Alfred Hospital, Institute of Academic Surgery, University of Sydney, 50 Missenden Road, Camperdown, Sydney, NSW 2050, Australia; General Surgery Department, Armidale Rural Referral Hospital, Hunter New England Health Services, 226 Rusden Street, Armidale NSW 2350, Australia; General Surgery Department, Armidale Rural Referral Hospital, Hunter New England Health Services, 226 Rusden Street, Armidale NSW 2350, Australia

**Keywords:** blunt abdominal and mesenteric trauma, non-operative management, delayed intestinal perforation, mesenteric ischaemia, case reports

## Abstract

Blunt bowel and mesenteric injuries (BBMI) are diagnostically challenging and may evolve despite apparent radiological resolution. This case describes biphasic small bowel perforation following non-operative management (NOM) after blunt abdominal trauma, highlighting limitations of conservative strategies and the need for prolonged vigilance. A 57-year-old woman sustained a Grade 4 small bowel perforation after a motor vehicle collision. She presented 7 days post-accident with abdominal pain but was haemodynamically stable and elected NOM. Interval computed tomography at 23 days demonstrated apparent resolution. At 21 weeks, she re-presented with crescendo abdominal pain, nausea, and vomiting. Imaging confirmed recurrent perforation at the original site. Laparotomy revealed a 3 cm full-thickness perforation adjacent to a chronic fibrotic stricture and mesenteric defect. Ileocolic resection was performed. Radiological resolution does not equate to definitive healing in high-grade BBMI. Trauma-induced chronic ischaemia may drive delayed stricture formation and late perforation, warranting structured long-term follow-up, and patient education.

## Introduction

Blunt bowel and mesenteric injuries (BBMIs) are rare, occurring in 1%–5% of blunt abdominal trauma cases [1, 2]. Their subtle presentation, coupled with polytrauma distractions, increases the risk of missed diagnoses, leading to delayed or inadequate management [3]. Up to 20% of hollow viscus injuries remain undetected on initial computed tomography (CT) scans, resulting in delayed intervention and increased mortality beyond 24 h [4]. While a liberal approach to laparotomy may reduce missed injuries, it carries significant morbidity without clear benefit [5].

This case highlights a biphasic small bowel perforation following blunt abdominal trauma, initially resolving with non-operative management (NOM) before re-perforating due to chronic ischaemic progression. It underscores the limitations of conservative management in high-grade injuries and the need for long-term surveillance, even in cases of apparent radiological resolution.

## Case presentation

A 57-year-old female presented to a remote hospital 7 days post-motor vehicle accident with a kangaroo at 80 km/h. The restrained driver had no loss of consciousness. Her medical history included hypertension, tubal ligation, and previous caesarean section. She reported worsening lower abdominal pain for the past 7 days. The clinical examination did not elicit any peritonism, nor signs of sepsis. It further revealed a seatbelt sign, which, in combination with non-specific abdominal tenderness, carries a 2.4-fold increased risk of bowel perforation [6].

Laboratory investigations showed C-reactive protein (CRP) of 335 mg/l, white cell count of 8.0 × 10^9^/l, haemoglobin of 117 g/l, and lactate of 1.2 mmol/l. Contrast-enhanced CT revealed free intra-abdominal and pelvic fluid, dilated small bowel loops, abrupt luminal calibre change, mesenteric stranding, and a localised perforation with an adjacent gas and fluid collection to the terminal ileum (Fig. 1a and b). Given clinical stability, delayed presentation, the patient opted for NOM with intravenous amoxicillin-clavulanic acid (1.2 g every 8 h for 3 days), thereafter transitioned to equivalent oral therapy for further 5 days. The patient was discharged on Day 5 from hospital. At Day 23 post-injury, our patient remained asymptomatic, continued to have normal gastro-intestinal functions and had a CT scan showing interval resolution of the stranding and perforated gas and fluid-filled collection in the small bowel mesentery, with no residual pelvic collection (Fig. 2a and b). At the time inflammatory markers have regrettably not been collected.

**Figure 1:**
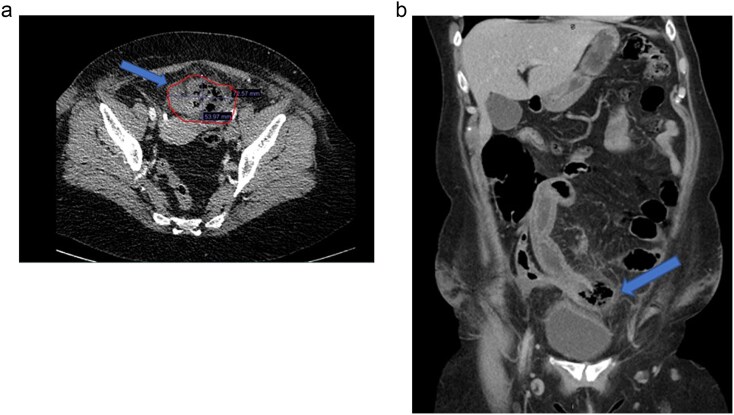
(a) Day 7 post motor vehicle accident. Axial contrast-enhanced CT scan: contained small bowel perforation 56 × 72 mm (blue arrow/red circled region). (b) Day 7 post motor vehicle accident. Coronal contrast-enhanced CT scan: small bowel rupture with contained perforation (blue arrow).

**Figure 2:**
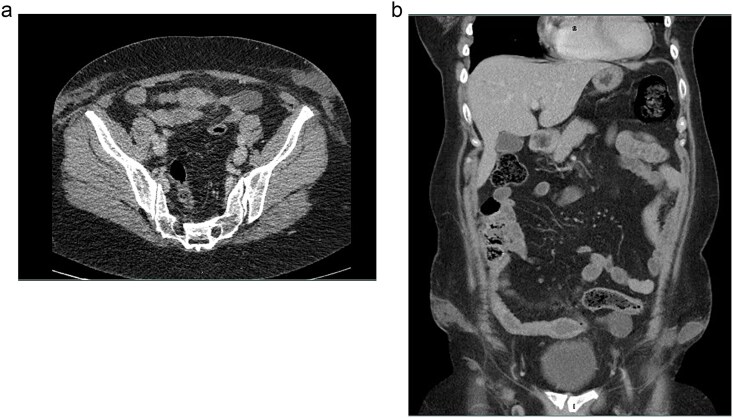
(a) Day 23 post motor vehicle accident. Axial contrast-enhanced CT scan: interval resolution of previous perforation. Nil collection. (b) Day 23 post motor vehicle accident. Coronal contrast-enhanced CT scan: interval resolution of previous perforation. Complete resolution of previous collection.

However, at Week 21 following the accident, she re-presented with a 3-day history of worsening left lower quadrant abdominal pain, nausea, and vomiting. Examination revealed mild tenderness without peritonism. CRP was elevated to 82 mg/l . The repeat CT (Fig. 3a and b) was consistent with a recurrent, contained small bowel perforation, comparable to findings on index CT scan.

**Figure 3:**
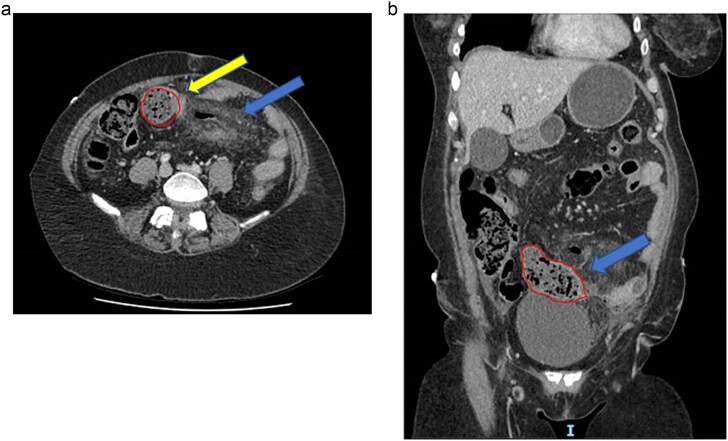
(a) Twenty-one weeks post motor vehicle accident. Axial contrast-enhanced CT scan: thickened terminal ileum (blue arrow) with localized perforation (red circle region), stricture distal to perforation (yellow arrow). (b) Twenty-one weeks post motor vehicle accident. Coronal contrast-enhanced CT scan: thickened terminal ileum (blue arrow) with localised perforation (red circle region).

At emergency laparotomy, a 3 cm full-thickness perforation at the mesenteric border of the distal ileum, 10 cm proximal to the ileocaecal valve, was identified, adjacent to a fibrotic stricture and a 50 mm mesenteric defect. An ileocolic resection with a stapled, side-to-side anastomosis was performed. Histology confirmed chronic ischaemic fibrosis, suggesting trauma-induced vascular compromise as the driver of this delayed perforation (Fig. 4).

**Figure 4:**
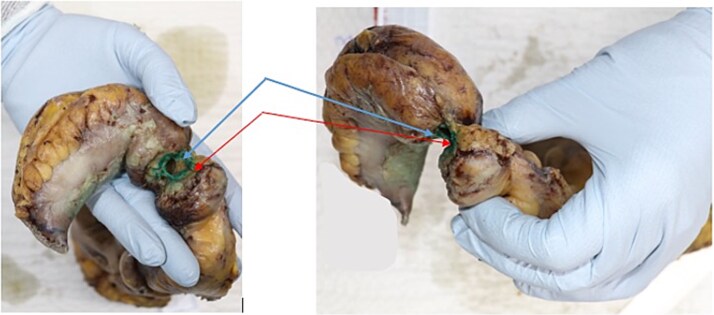
Histopathology of ileo-caecal resection specimen: terminal ileum perforation (blue arrows), distal stricture (red arrow).

## Discussion

BBMIs pose significant diagnostic challenges due to their low incidence, subtle clinical signs, and imaging limitations [6]. This case highlights the potential for interval radiological resolution to mask evolving ischaemia, ultimately leading to delayed perforation. While NOM is appropriate for Grade 1–2 BBMIs, for higher grade injuries (Grade 3–4) surgery is recommended. In hindsight, despite the patient’s preference for NOM at index presentation, optimal management was not achieved.

CT, despite being the gold standard, misses up to 20% of hollow viscus injuries, leading to delayed diagnoses and increased morbidity with each hour of delay [6]. This case emphasises that radiological resolution does not equate to definitive healing, particularly in the context of trauma-induced mesenteric ischaemia. Progressive fibrosis and ischaemic necrosis may culminate in late perforation, even after apparent recovery.

Recent data suggest that delayed diagnosis of BBMI remains a significant challenge despite advanced imaging, prompting investigations of predictive tools to reduced missed injuries and therefore mitigate adverse outcomes [7].

## Conclusion

This case highlights pitfalls of NOM in BBMIs, particularly in rare cases like ours, where patient-led, non-standard care was pursued with falsely reassuring imaging and short-lasting resolution, prior cumulating in delayed, ischaemia-induced intestinal perforation. While interval CT resolution may provide reassurance, it does not preclude ongoing mesenteric ischaemia and late perforation risk. Trauma clinicians should maintain high clinical suspicion, ensure structured follow-up, and provide clear patient education to mitigate the risks of delayed deterioration. Future studies should explore the feasibilities of alternative radiological imaging modalities and predictive tools that may offer greater sensitivity in this subset of trauma patients.
